# Supplementary material for: How does ChatGPT-4 preform on non-English national medical licensing examination? An evaluation in Chinese language
Source: PLOS Digit Health. 2023 Dec 1;2(12):e0000397. doi: 10.1371/journal.pdig.0000397 (PMC10691691; doi:10.1371/journal.pdig.0000397)
Supplement: S1 Table — (DOCX) [file pdig.0000397.s001.docx]

**S1 Table: The original question**

| Unit 1 | https://drive.google.com/file/d/1aLFag7Z40khalnKckfZR6DYp2TRjWy46/view?usp=share_link |
| --- | --- |
| Unit 2 | https://drive.google.com/file/d/1xElTkdJ8y33WSAmOq0voezaxkTg9OXKP/view?usp=share_link |
| Unit 3 | https://drive.google.com/file/d/1gkbdWjw1imp2OI-AIvs8Ycp1KcH_iiPZ/view?usp=share_link |
| Unit 4 | https://drive.google.com/file/d/1NU1MvFMm4d42zMNpmUlyMk7yyHuWly5x/view?usp=share_link |
