# Supplementary material for: How does ChatGPT-4 preform on non-English national medical licensing examination? An evaluation in Chinese language
Source: PLOS Digit Health. 2023 Dec 1;2(12):e0000397. doi: 10.1371/journal.pdig.0000397 (PMC10691691; doi:10.1371/journal.pdig.0000397)
Supplement: S2 Table — (DOCX) [file pdig.0000397.s002.docx]

**S2 Table：Adjudication criteria for accuracy, concordance.**

| Accurate:1. Provide the answer accurately.  2.When the judge determines that there is not a unique answer, the AI outputs multiple choices, among which contains the correct answer, and the other options are also completely correct. | Concordant: Explaining affirms the answer |
| --- | --- |
| Inaccurate 1. No answer is provided  2.An incorrect answer is provided  3.Multiple answers are provided, among which there is an incorrect answer, even if the correct answer is included. | Discordant: Explanations are contradictory. |
| Indeterminate: 1.AI output is not a single answer election.  2.When the judge determines that there is a unique answer, AI output provides multiple choices.  3.AI believes that there is not enough information." |  |
