# Supplementary material for: How does ChatGPT-4 preform on non-English national medical licensing examination? An evaluation in Chinese language
Source: PLOS Digit Health. 2023 Dec 1;2(12):e0000397. doi: 10.1371/journal.pdig.0000397 (PMC10691691; doi:10.1371/journal.pdig.0000397)
Supplement: S3 Table — (DOCX) [file pdig.0000397.s003.docx]

**S3 Table：Kappa statistic for interrater agreement between adjudicating physicians.**

|  | Accuracy | | Concordance | |
| --- | --- | --- | --- | --- |
|  | Cohen κ | n | Cohen κ | n |
| Unit 1 | 1 | 42 | 1 | 0.88 |
| Unit 2 | 0.88 | 68 | 1 | 68 |
| Unit 3 | 0.83 | 71 | 0.97 | 71 |
| Unit 4 | 0.85 | 79 | 1 | 79 |
