# Supplementary material for: How does ChatGPT-4 preform on non-English national medical licensing examination? An evaluation in Chinese language
Source: PLOS Digit Health. 2023 Dec 1;2(12):e0000397. doi: 10.1371/journal.pdig.0000397 (PMC10691691; doi:10.1371/journal.pdig.0000397)
Supplement: S4 Table — (DOCX) [file pdig.0000397.s004.docx]

**S4 Table：Crosstab for evaluating translation effectiveness**

|  | |  | | Sum |
| --- | --- | --- | --- | --- |
|  |  | Accurate | Inaccurate |  |
|  | Original questions | 197 | 63 | 260 |
|  | Translated questions | 201 | 59 | 260 |
| Sum | | 398 | 122 | 520 |
